# Challenges of access to care for children with cancer living in the Gaza Strip/occupied Palestinian territory in 2022: a cross-sectional study

**DOI:** 10.1136/bmjgh-2024-015493

**Published:** 2026-01-07

**Authors:** Salwa Massad, Mervett Isbeih, Khalid Abu Saman, Margarida B Goncalves, Lamia Mahmoud, Nasim Pourghazian, Giuseppe Troisi, Zeena Salman, Sima Jeha, Shannon Barkley, Richard Peeperkorn

**Affiliations:** 1 World Health Organization, Jerusalem, State of Palestine; 2 World Health Organisation Regional Office for the Eastern Mediterranean, Cairo, Egypt; 3 St Jude Children’s Research Hospital, Memphis, Tennessee, USA

**Keywords:** Global Health, Child health, Cancer, Health services research, Public Health

## Abstract

**Introduction:**

Cancer care in humanitarian settings is very challenging, and patients may face significant barriers to accessing the care they need. This study explored access to advanced diagnostic and treatment services for children with cancer in humanitarian settings, taking Gaza as a case study.

**Methods:**

The study was based on 51 key informant interviews and two focus group discussions with close relatives of children with cancer, healthcare providers and Ministry of Health officials between November 2021 and January 2022. We also analysed referral data for paediatric oncology care outside Gaza in 2021.

**Results:**

Among the structural barriers to cancer care are complex and lengthy referral mechanisms, along with an unclear permit system. These challenges contribute to significant delays in both diagnosis and the initiation of treatment. The referral pathway involves multiple administrative and logistical steps to secure approval for treatment outside Gaza. It begins with a physician-initiated referral, approval of the Ministry of Health Service Purchasing Unit, and concludes with exit permit requests for the child and a companion, which must be approved by the Israeli Gaza Coordination and Liaison Administration. Analysis of 2021 referral data reveals that 25% of children with cancer experienced permit delays of over 1 month, and 8% died while waiting for an exit permit.

**Conclusions:**

The urgent need to scale up cancer care in Gaza is critical, particularly for children who face severe challenges due to ongoing conflict, the Israeli blockade since 2006 and the closure of the only paediatric oncology department. Immediate, coordinated national and global efforts are essential to overcome political, medical and financial barriers. Improving health outcomes and survival for children with cancer in Gaza requires addressing the root causes of late diagnosis, as well as the complex referral and unclear permit processes that delay timely access to specialised care.

**WHAT IS ALREADY KNOWN ON THIS TOPIC**

- Cancer care in humanitarian settings is very challenging, and patients may face significant barriers to accessing the care they need.

**WHAT THIS STUDY ADDS**

- There is a scarcity of literature on access to oncology services in humanitarian settings, particularly paediatric oncology. In this study, we have illustrated a thorough picture of access to paediatric oncology services and the referral process and its challenges in one of the worst existing humanitarian settings.

**HOW THIS STUDY MIGHT AFFECT RESEARCH, PRACTICE OR POLICY**

- This study illustrates the pathway of a child from time of diagnosis to the result of request for a permit to access healthcare outside of Gaza. We identify barriers within the healthcare system, and specifically, the referral system that, in return, allows for establishment of a baseline for future works to be conducted.

## Introduction

 Globally, over 1000 children under 20 are diagnosed with cancer every day. Unfortunately, their chance of survival hinges heavily on where they live. In high-income, stable countries with well-developed healthcare systems, more than 80% of children with cancer have a good chance of surviving. However, for children in low-income and middle-income countries (LMICs), their survival rate plummets to just 15%–45%. This disparity stems from a complex interplay of factors including under-resourced healthcare systems and limited political prioritisation of childhood cancer.^1^

Patients with cancer represent a particularly vulnerable group during emergencies due to the complex ethical and practical challenges involved in delivering treatment. Many cancer medications require specialised service delivery systems and are frequently given intravenously in hospital settings over several days. In many LMICs, the capacity to provide cancer care is often already constrained before any emergency occurs.^2^ Access to cancer care is a fundamental pillar, yet it is frequently reported as a major barrier, with geopolitical factors further restricting patients’ rights to diagnostic and therapeutic services.^3^ For example, a qualitative study of patients with cancer and their families in Sulaymaniyah, Iraq, found that the challenges patients faced in obtaining care include having to travel to multiple hospitals across different cities due to shifting security conditions, selling personal assets to finance treatment and struggling to obtain medications from private pharmacies and/or the black market.^4^ Similarly, in Syria, access to specialist physicians and cancer treatment was severely limited in both government-controlled and besieged areas.^5^

**Figure 1:**
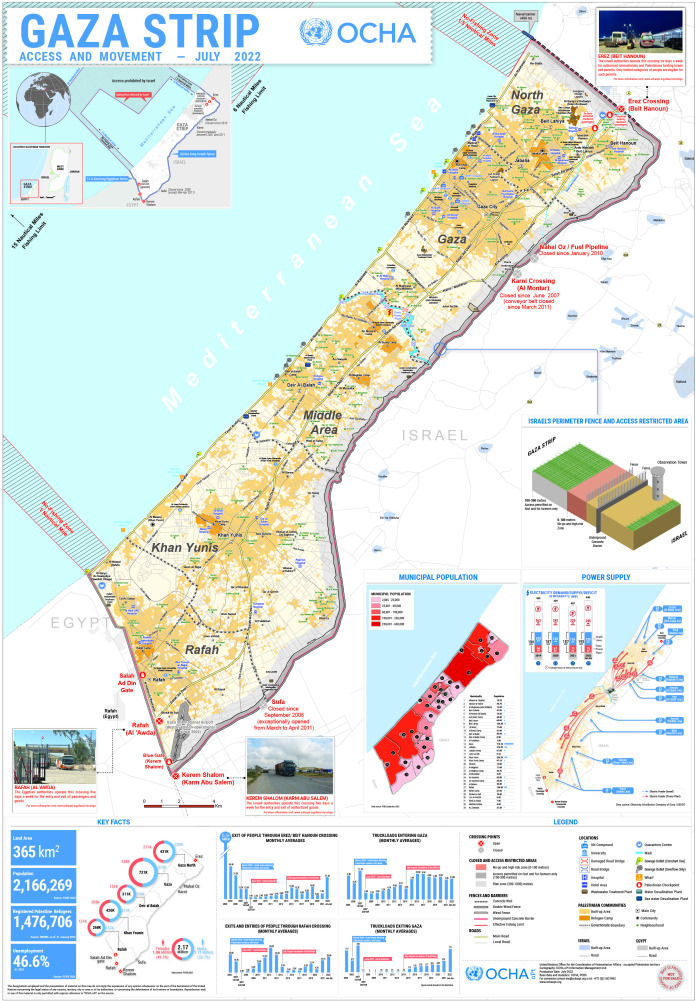
Gaza closure map, United Nations Office for the Coordination of Humanitarian Affairs (OCHA).^22^

The situation in the Gaza Strip is uniquely challenging. Since 2006, the Israeli blockade has imposed a protracted humanitarian crisis, restricting movement through only two entry points: Erez in the north, requiring Israeli-issued permits, and Rafah in the south, controlled by Egypt (figure 1).^6^ The blockade has pushed Gaza’s healthcare system to the brink of collapse, with critical shortages of essential medications, equipment and healthcare personnel. Patients frequently face denial of permits needed to seek life-saving treatment abroad.^7^

Children with cancer in Gaza face particularly acute challenges. Timely and accurate diagnosis, along with access to multidisciplinary subspecialty care, is critical for successful treatment outcomes. Before 7 October 2023, Gaza had two governmental oncology hospitals: the Rantisi Pediatric Oncology Hospital, supported by the Palestine Children’s Relief Fund (PCRF) (social work, child life, emergency drug stock, etc), and the Turkish Palestinian Friendship Hospital for adults. Boys were transferred to the adult hospital at age 14, girls at 16. Approximately 210 children were diagnosed with cancer annually in Gaza before October 2023.^8^ However, care was hindered by frequent drug shortages, limited diagnostic equipment and the absence of radiotherapy services. Chronic political instability and lack of secured funding resulted in 30%–40% of essential chemotherapy drugs being out of stock at any time, causing treatment delays and poorer prognoses.^6^ A study of 131 children diagnosed with acute lymphoblastic leukaemia from 2010 to 2015 showed that all required at least one referral abroad due to inadequate diagnostics, inconsistent chemotherapy access and insufficient supportive or intensive care; over half required multiple referrals for additional treatments such as radiation and bone marrow transplant.^9^

All Palestinians in the Gaza Strip needing to leave Gaza require Israeli-issued permits.^10^ Between 2018 and 2021, 43% of children approved to travel for hospital appointments outside Gaza did not have the approval of one of their parents as a companion.^11^ In 2022, 33% (6500/20 295) of the permit applications for patients from Gaza were delayed (more than 30 days of the application for a permit), with over one-third (35%) of these related to patients with cancer.^12^

However, since 7 October 2023, Israeli attacks targeting hospitals have severely endangered thousands of vulnerable individuals in Gaza, including patients with cancer, those with chronic diseases, pregnant women and newborns. The Israeli blockade, which has cut-off humanitarian supplies, has further exacerbated this dire situation.^13^ Fuel shortages combined with ongoing Israeli strikes forced the closure of Gaza’s only paediatric oncology department at Al-Rantisi Hospital and the last remaining cancer treatment centre, leaving countless children and other patients without access to life-saving care. This catastrophic collapse of the healthcare system has resulted in a humanitarian crisis, with many patients facing a slow death due to lack of treatment and medical resources.^14^

Literature on access to oncology services in humanitarian settings, particularly paediatric oncology in the Gaza Strip, is limited. To address this knowledge gap, this study examined access to advanced diagnostic and treatment services for children with cancer in humanitarian contexts, using Gaza as a case study.

## Methods

### Study design

The study used a mixed-methods approach. Authors conducted 51 semistructured interviews with close relatives of children with cancer, healthcare providers, Ministry of Health (MoH) focal points in both the West Bank and Gaza, and the PCRF (table 1). We asked physicians to nominate parents of children with cancer while ensuring a balanced male to female ratio of children during recruitment.

**Table 1:** Overview of the key informant interviews with stakeholders, N=51, 2021

Representative	Interviewee/participant	Number of interviews
Oncology hospitals in Gaza		12
	CEO of Turkish hospital	1
	Oncologists	4
	Pathologist	1
	Head nurses	3
	Nurses	3
Oncology hospitals in the West Bank (Bet Jala/Bethlehem, Al-Najah/Nablus, Augusta Victoria Hospital/Jerusalem)		7
	Oncologists	4
	Head nurses	3
Ministry of Health-Ramallah		2
	General Director of Procurement	1
	Director of Central Drug Store	1
Ministry of Health-Gaza		12
	Deputy Minister of Health	1
	General Director of Pharmacy	1
	Deputy Director of Pharmacy	1
	Hospital pharmacists	2
	Head of Primary Healthcare and Hospital Pharmacy Department	1
	Director of Referral Abroad Department	1
	Director of Procurement	1
	Director of the Drugs and Pharmaceutical Department	1
	Deputy Director of the Drugs and Pharmaceutical Department	1
	Director of Central Drug Store	1
	Head of Liaison Office	1
The Palestine Children’s Relief Fund		4
	General Director	1
	Paediatric oncologist	1
	Project coordinator	1
	Social worker	1
Parents/relatives of children with cancer		14
	Mothers	9
	Fathers	3
	Grandmother	1
	Aunt	1

In addition, two focus group discussions were conducted: One was conducted with nine mothers of children with cancer at Rantisi hospital (Gaza), and another with four healthcare providers in Gaza (two paediatric oncologists, a hospital pharmacist and a pathologist). Following the interviews and the focus group discussion with carers of children with cancer, the authors conducted two technical meetings with healthcare providers in oncology hospitals in both Gaza and the West Bank, and another meeting with the MOH focal points in Gaza to delve deeper into the referral process for children with cancer, as described by their caregivers.

Interviews and focus group discussions were conducted between November 2021 and January 2022. Following data analysis, a validation workshop was conducted with all interviewed MOH focal points including healthcare providers to discuss and validate study findings.

To further support and contextualise the qualitative findings on referrals and barriers to cancer care, and to provide essential empirical evidence through data triangulation, the authors analysed two referral datasets (1) requests for referral data for all children with cancer (aged 0–12 years) in 2021 from the paediatric oncology department at Rantisi hospital and (2) 2021 referral data for all children with cancer (aged 0–17 years) from the Referral Abroad Department and the Cancer Registry at the Palestinian Health Information Center-Gaza.

Oral consent was obtained from all study participants following a description of the study. Once transcription was completed, all recordings were deleted. All data were anonymised prior to analyses to protect confidentiality. Fourteen interviews were conducted with the relatives of children with cancer between the ages of two and 17. Five of the children were girls and nine were boys, and their ages were between one and 15 years. A focus group was conducted with nine mothers of children with cancer; seven of the children were boys and two were girls. Among the children, five had leukaemia, one had Langerhans Cell Histiocytosis, one had idiopathic thrombocytopenic purpura, one had medulloblastoma and one had Wilms tumour.

### Patient and public involvement

It was not possible to involve patients’ caregivers or the public in the design, conduct, reporting or dissemination of our research. This was primarily due to a lack of direct access to caregivers of children with cancer, as participants were recruited by paediatric oncologists following interviews with these clinicians. Additionally, the urgency of conducting research in a conflict zone did not allow sufficient time for public involvement. However, paediatric oncologists were involved in the study design, reporting and dissemination plans, and the results were shared with the MOH to inform policy decisions.

### Qualitative measurements and outcomes

Key questions for interviews and focus group discussions were developed based on WHO CureAll Framework,^1^ WHO National Cancer Assessment Report 2019.^15^ Key questions included the perceived barriers to paediatric oncology services in Gaza, referral pathways of children with cancer and recommendations to strengthen paediatric oncology services in Gaza.

### Quantitative Study outcomes

Referral pathway for children with cancer: Analysed from the point of the attending oncologist’s request for the child’s referral to the child accessing care at the referral hospital.

Outcome of referrals of children with cancer: Studied based on referral data for children below 18 with cancer in 2021. The examined referral outcomes included the status of each application (whether the paediatric oncologist’s referral request for a child with cancer was approved by Israeli authorities, delayed for more than 30 days before approval or denied), the number of referrals per child, and deaths among children referred outside Gaza for advanced care.

### Data analysis

#### Qualitative data

Following transcription, initial coding broke the data down to themes and subthemes. Within each of the formal codes, subcodes were developed to indicate the location of discussion (Gaza, West Bank). The subcoded text was then summarised with quotations and the frequency of each subcode reported by the number of individual interviews.

#### Quantitative data

For the quantitative analysis of referral data, we used descriptive statistics to estimate means and percentages using R Studio software V.3.6.3. The available referral data included date of referral, place of referral, reason for referral, and diagnosis of the patient’s case and outcome of referral.

### Study findings

#### Complex and delayed referral mechanisms

**Figure 2:**
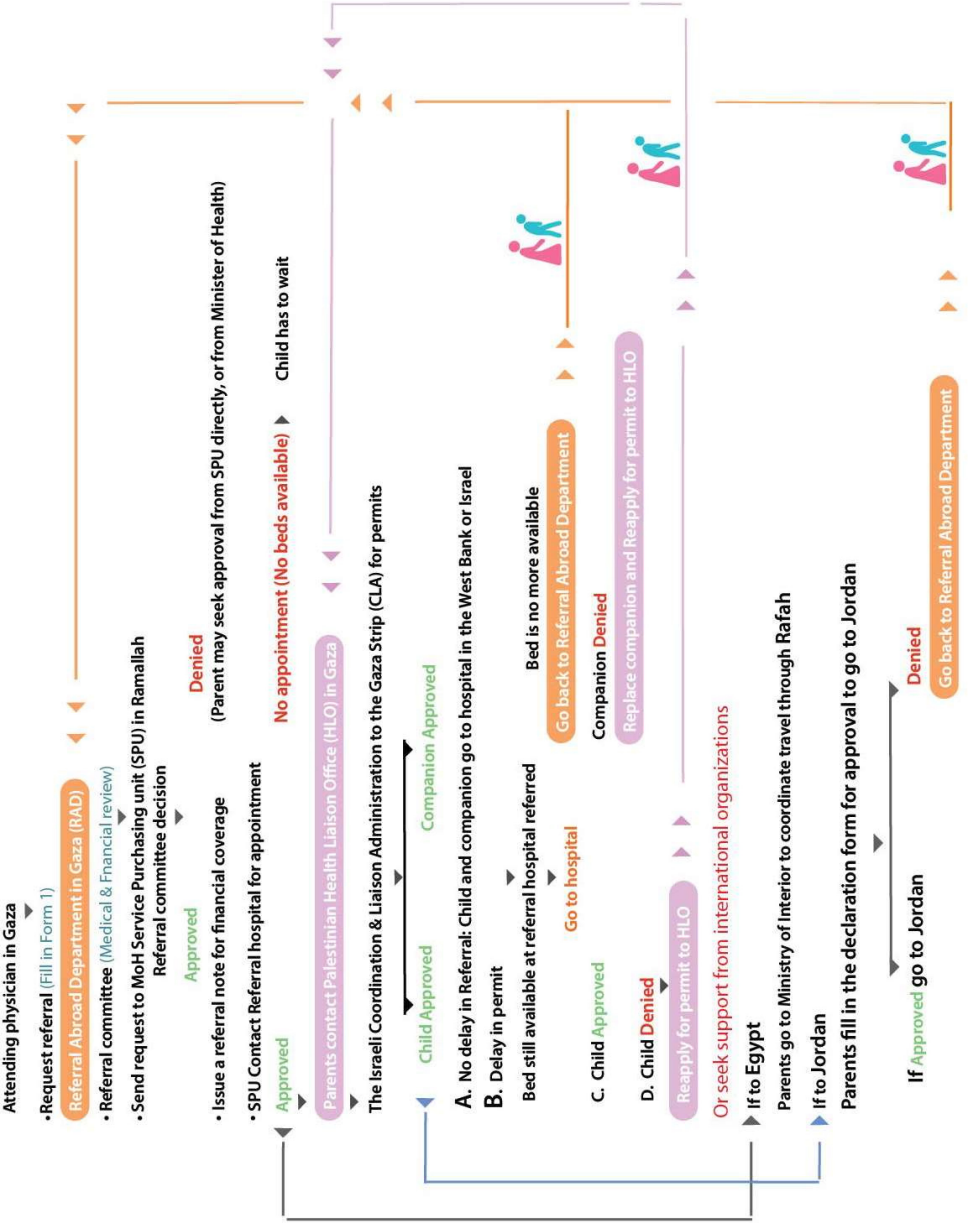
Referral pathways for children with cancer.

Due to permit delays and uncertainty surrounding processing times and approvals, the diagnosis and initiation of treatment for children with cancer in Gaza can take several months. Based on interviews and focus group discussions, the authors developed a referral pathway (figure 2) illustrating the multiple steps a child diagnosed with cancer must navigate to secure a referral for diagnosis or treatment outside Gaza.

The process begins when the attending physician in Gaza completes a ‘Form 1’ request for referral and submits it to the Referral Abroad Department. This department convenes a committee responsible for the medical and financial review of requests. Once approved, the referral is forwarded to the MiOH Service Purchasing Unit (SPU) in Ramallah/West Bank. If the SPU denies the request, parents may appeal directly to the SPU or the Minister of Health.

If SPU approves the request, it issues a referral note for financial coverage and contacts the referral hospital in the West Bank, Jordan, Egypt or Israel to schedule an appointment. If no beds are available, the child must wait. When beds are available, parents then apply for exit permits for the child and a companion through the Palestinian Health Liaison Office (HLO), which submits the permit to the Israeli Gaza Coordination and Liaison Administration (CLA) for approval. Some children must navigate this complex referral process every 2 weeks to maintain their chemotherapy schedules.

#### Outcomes of CLA permit requests to exit Gaza for treatment

Permit requests submitted to the CLA to exit Gaza for medical treatment can result in four possible outcomes (see figure 2):

Scenario A: Child and companion are approved without delay, allowing timely travel to the referral hospital outside Gaza.

Scenario B: Child and companion are approved, but permit approval is delayed—often exceeding 1 month. Based on the study findings, delays arise from multiple factors, including late referral requests by attending physicians, slow issuance of referral notes by the Palestinian MMOH SPU in Ramallah/West Bank, lack of financial coverage, unavailability of hospital beds and repeated permit denials or delays for medical companions.

According to MoH referral data, 271 children under 18 with cancer were referred outside Gaza in 2021. Of these, 67 experienced delayed approvals (over 1 month), one was denied, and 21 (7.8%) died while awaiting permits, 14 were male and 7 females, with a median age of 10 years. Among the deceased, 13 had delayed referrals.

Hospital data show 563 referral requests for 217 children under 13, half under 7 years old, in 2021. Many had multiple referrals (1–16 per child), with a median interval of 4 days between requests, ranging from simultaneous to over 9 months apart. Among these children, 11 (5.1%) died in 2021; 6 had referral delays exceeding 30 days.

Delays often cause children to lose reserved hospital beds, forcing the Referral Abroad Department to resubmit requests to SPU. For referrals to King Hussein Cancer Center in Jordan, parents must also complete a declaration form for entry permission. If denied, they return to the Referral Abroad Department. For referrals to Egypt, parents coordinate with Gaza’s Ministry of Interior to issue passports and arrange travel through the Rafah crossing.

We would make an appointment, but the Israelis would take 20 days to respond to our permit request. Sometimes they said our request was under investigation, so we missed the appointment. We would make another appointment and request another permit, which took another month. They only approved our request after applying multiple times.

-Interview with the mother of a boy with cancer, November 2021

By the time we got the permit to go to Al-Makased hospital [East Jerusalem] for surgery, the tumor grew so big that it broke her leg.

-Interview with the mother of a girl with cancer, November 2021

We were supposed to go to Augusta Victoria [East Jerusalem] because my son needed radiation, but we were delayed for one month. During that one month delay, my son’s stage of illness worsened from stage one to stage three.

*-* Interview with the mother of a boy with cancer, November 2021

The first time we applied for a referral for my grandson, we got the permit on time and he was treated. The second time, there was a delay, and he died before the permit was approved.

-Interview of a grandmother of a boy with cancer, November 2021

Scenario C: The child is approved, but the companion (usually the mother) is denied a permit. Families must select an alternative companion and reapply through the Palestinian HLO.

We applied for permits for me and his father but were refused. Then we applied for his grandmother, who died while waiting for approval. Finally, my husband’s cousin applied and was approved to accompany my son*.*

-Interview with the mother of a boy with cancer, November 2021

Scenario D: The child is denied a permit outright. Reasons for denials are often unclear to families and healthcare providers. Parents usually seek assistance from international humanitarian organisations to appeal.

## Discussion

The WHO’s Global Initiative for Childhood Cancer emphasises that timely diagnosis, early referral and treatment initiation are critical drivers of survival, aiming to begin treatment within approximately 30 days of diagnosis in paediatric oncology.^1^ Clinical evidence supports this benchmark: a 2023 study in Ethiopia reported that children with acute lymphoblastic leukaemia who commenced chemotherapy 30–90 days post-diagnosis had a significantly higher induction mortality rate than those treated earlier.^16^ In our study, nearly 25% of referred paediatric cancer patients in Gaza experienced delays exceeding 30 days, indicating systemic failures in meeting international standards and likely contributing to preventable morbidity and mortality.

Delayed access to even basic healthcare necessities threatens the chances for survival for those with fragile health conditions. In line with a previous study in Syria, the study showed inhibited access to management options in besieged Gaza compared with the West Bank, in terms of radiation therapy, bone marrow transplantation and positron emission tomography scans.^5^ Similarly, in Iraq—another conflict-affected and resource-constrained setting—referral system challenges caused delays due to security issues, costs and poor communication between healthcare providers and referral facilities.^17^

This study provides a comprehensive picture of paediatric oncology access and referral challenges in one of the world’s most severe humanitarian crises. Pitfalls in Gaza’s stagnated referral system greatly impact health outcomes for children with cancer. Nearly a quarter of patients encountered permit delays in 2021, echoing a long-standing crisis documented in a 2008–2017 study where 23% of patients with cancer faced delays in approvals (Bouquet *et al*). WHO data further underscore this issue, showing that between January and December 2022, delays in approval for all patients ranged from 16% to 40%.^18^ For paediatric cancer patients, missing even a single chemotherapy dose or cycle can dramatically reduce treatment efficacy.^6^ A WHO study analysing outcomes of children in Gaza denied permits for referral to the West Bank and Jordan between 2015 and 2017 found higher mortality among those with denied permits, with a HR of 1.45 (95% CI 1.19 to 1.78, p<0.001), after adjusting for age, sex, procedure type and cancer type.^19^

The urgent need to scale up cancer care in Gaza cannot be overstated. Immediate coordinated national and global efforts are required to overcome the political, medical and financial obstacles faced by patients with cancer. The situation for children with cancer in Gaza is critical—they require urgent humanitarian assistance and access to specialised care that is increasingly out of reach due to ongoing conflict and logistical challenges.^20^ The war has caused a catastrophic breakdown of cancer care services,^21^ leaving thousands without treatment options and worsening an already dire humanitarian situation.

Children with cancer require continuous treatment, including chemotherapy and radiotherapy, both severely limited due to the siege and conflict. Radiation therapy has been inaccessible for years, necessitating transfers outside Gaza for treatment—currently impossible due to border closures. Many essential chemotherapy medications, both intravenous and oral, are unavailable; only about 20% of oncology medications and adjuvant therapies are in stock, alongside severe shortages of palliative care drugs. The current conflict has further compounded these shortages, making timely diagnosis and treatment increasingly difficult. Diagnostic services such as CT scans and MRIs are also hindered by lack of necessary resources. At the time of writing, Gaza had only one hospital with an oncology department in the south and one outpatient cancer clinic in Gaza City. On average, 30 children receive outpatient treatment daily, while about 12 are hospitalised at any time.

Most patients with cancer require treatment abroad; however, the war and border closures have severely limited their access to care. Although children with cancer have been prioritised for medical evacuation, the process has been slow, especially after the Rafah crossing closure. From June 2024 to June 2025, of 2423 evacuated patients, only 642 were cancer patients, including 140 children. Currently, approximately 500 children with cancer endure dire health conditions exacerbated by ongoing conflict and urgently need evacuation. Between June and December 2024, among 378 evacuated patients, only 165 were cancer patients, including 66 children. Approximately 700 children with cancer currently face critical health challenges worsened by conflict and require urgent medical evacuation. These children suffer mainly from lymphatic and blood cancers and face a high risk of death without proper treatment and follow-up. The ongoing conflict has severely restricted medical supplies and damaged healthcare infrastructure, leading to a significant decline in cancer treatment availability and a halt in new diagnoses.^21^

To the best of the authors’ knowledge, this is the first qualitative study exploring referral pathways and associated barriers for children with cancer seeking care outside Gaza. The study has standard limitations of non-probability sampling, relying on self-reporting subject to recall or social desirability bias. However, these were mitigated through data triangulation involving caregivers, healthcare providers and referral datasets. Validation workshops with MOH focal points and providers further strengthened the findings by contextualising and confirming emerging themes.

Cancer care for children in humanitarian settings is complex and challenging. Children often require long-term, complex treatment that is difficult to access in disrupted health systems. Gaza’s challenges—blockade, permit requirements and political complexities—intensify barriers common to conflict zones. Nonetheless, lessons from Gaza, including the need for coordinated referral systems and international advocacy to overcome bureaucratic and political obstacles, are broadly relevant. Future research should focus on supportive and palliative care needs, such as psychosocial support, pain management and survivorship care, which remain underexplored in conflict settings.

Our study emphasised that there is an urgent need to address the root causes of the late diagnosis and the complex referral process in Gaza. While rebuilding a war-torn healthcare system takes time and sustained effort, by supporting local talent, building essential infrastructure, providing ongoing support and negotiating to ease the restrictive measures preventing children with cancer from seeking care outside of Gaza, we can help create a brighter future for children with cancer in Gaza and their families.

## Data Availability

Data are available on reasonable request.
